# Genetics of Tinnitus: Time to Biobank Phantom Sounds

**DOI:** 10.3389/fgene.2017.00110

**Published:** 2017-09-04

**Authors:** Christopher R. Cederroth, Anna K. Kähler, Patrick F. Sullivan, Jose A. Lopez-Escamez

**Affiliations:** ^1^Experimental Audiology, Department of Physiology and Pharmacology, Karolinska Institutet Stockholm, Sweden; ^2^Department of Molecular Epidemiology and Biostatistics, Karolinska Institutet Stockholm, Sweden; ^3^Department of Genetics, University of North Carolina, Chapel Hill NC, United States; ^4^Department of Psychiatry, University of North Carolina, Chapel Hill NC, United States; ^5^Otology & Neurotology Group, Department of Genomic Medicine, Pfizer-Universidad de Granada-Junta de Andalucía Centre for Genomics and Oncology Research (GENyO) Granada, Spain; ^6^Department of Otolaryngology, Instituto de Investigación Biosanitaria ibs.GRANADA, Hospital Virgen de las Nieves, Universidad de Granada Granada, Spain

**Keywords:** tinnitus, genetics, heritability, subtype, neuropsychiatry, GWAS (genome-wide association study), whole exome sequencing

## Abstract

Tinnitus is a common phantom sensation resulting most often from sensory deprivation, and for which little knowledge on the molecular mechanisms exists. While the existing evidence for a genetic influence on the condition has been until now sparse and underpowered, recent data suggest that specific forms of tinnitus have a strong genetic component revealing that not all tinnitus percepts are alike, at least in how they are genetically driven. These new findings pave the way for a better understanding on how phantom sensations are molecularly driven and call for international biobanking efforts.

## Perspective

For decades, tinnitus was considered a consequence of environmental factors, with low genetic contribution. The numerous etiologies, such as aging (presbycusis), noise exposure, stress, hypertension, diabetes, ototoxic medications, temporomandibular joint disorders, traumatic or ischemic damage, vascular problems, middle-ear problems, and the complex pathophysiology involving peripheral and central auditory and non-auditory structures, have led to the belief that tinnitus is a consequence of some other disease.

The knowledge on the genetic basis of tinnitus was recently reviewed (Vona et al., 2017) and phenotyping strategies have been proposed based on the assumption that tinnitus should be considered as an ensemble of sub-entities called subtypes (Lopez-Escamez et al., 2016). A small familial aggregation study (*n* = 198 families) found no obvious heritability (Hendrickx et al., 2007), and the first large population-based family study (*n* = 52,045) made an estimate of heritability of 0.11 (Kvestad et al., 2010). But a recent twin study revealed a higher heritability of 0.4, indicating that a larger fraction of the variance can be due to genetic variants than previously reported (Bogo et al., 2016). Such discrepancies may originate from differences in the design and formulation of the questionnaires, which have been found to vary greatly in prevalence studies on tinnitus (McCormack et al., 2016).

The tinnitus phenotype could also be grounds for diverging heritability values, but what defines a tinnitus phenotype is highly debated. We indeed consider that a more precise definition of a homogeneous phenotype will be essential in the design of genetic studies. A larger twin study performed by members of the TINNET^1^ consortium recently considered the laterality of tinnitus as a potential genetic subtype (Maas et al., 2017). A key finding was that bilateral tinnitus had higher heritability than unilateral tinnitus. The study was based on self-reported data from the Swedish Twin Registry, one of the largest twin registries in the world (Lichtenstein et al., 2002, 2006; Pedersen et al., 2002; van Dongen et al., 2012). Of a total of 70,186 twins that answered questions related to tinnitus, 15% of them experienced tinnitus. 10,464 concordant or discordant pairs for tinnitus were identified, in which 6,990 subjects had tinnitus. When considering tinnitus as a whole, a moderate genetic contribution (near 40%) was found (Bogo et al., 2016). However, when twins were stratified – based on tinnitus experienced in one ear (unilateral) or in both ears (bilateral) as well as on gender – bilateral tinnitus reached a heritability of 0.68 in men (Maas et al., 2017). Such values are close to the levels of heritability for schizophrenia and attention deficit hyperactive disorder (ADHD), two well known heritable conditions (**Table 1**). Although more work is required for establishing the contribution of hearing loss in such high heritability values (e.g., by including exhaustive auditory data), these findings open the possibility of specific forms of tinnitus being more genetically driven than others and pave the way for future genetic studies considering subtypes. These findings, however, need to be replicated in other twin cohorts as well as familial studies.

**Table 1:** Classification of tinnitus heritability against other disorders.

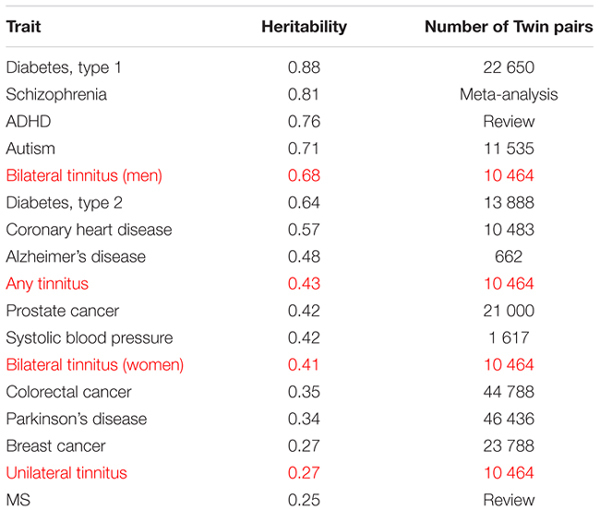

*Modified from van Dongen et al. (2012) with permission from the Nature Publishing Group. Tinnitus values are marked in red.*

In line with genetic association studies of other complex traits, published studies to find genetic markers for chronic tinnitus patients in candidate genes have been underpowered (*n* = 95–288) and failed to identify robustly associated genetic variants (Sand et al., 2010, 2011, 2012a,b; Gallant et al., 2013). In spite of a lowly powered tinnitus group (*N* = 167) and no significant associations found, a recent genome-wide association study (GWAS) identified some pathways (e.g., oxidative stress, endoplasmatic reticulum stress, and serotonin reception mediated signaling) potentially involved in tinnitus (Gilles et al., 2017). Supporting the need of better characterizing the tinnitus cases, Pawelczyk and colleagues investigated 99 single nucleotide polymorphisms targeting 10 genes involved in the potassium recycling pathway in the inner ear (128 tinnitus cases and 498 controls both exposed to occupational noise) (Pawelczyk et al., 2012). However, two of the identified SNPs were not subjected to multiple testing and were thus considered nominally significant. An important lesson from GWAS on other complex traits, such as schizophrenia and major depressive disorder (Sullivan et al., in press), is that far larger sample sizes are needed in order to identify genome-wide significant genetic variants. Therefore, an important next step in the search for genetic variants associated with tinnitus will be to perform joint GWAS analysis of thousands of tinnitus patients and healthy controls.

Since familial tinnitus is a rare condition, the selection of multiplex tinnitus families, in addition to unrelated cases and controls, for exome sequencing, is another potential strategy to be used for the discovery of genes involved in tinnitus. This strategy has been successful in the identification of *DTNA*, *PRKCB*, *SEMA3D* and *DPT* in autosomal dominant Meniere disease (Requena et al., 2015; Martin-Sierra et al., 2016, 2017).

With tinnitus being a condition with highly unmet clinical needs (Cederroth et al., 2013), the recent identification of a high heritability opens door to exciting research. Since it is more than likely that tinnitus is a polygenic trait and it will require the study of several thousand samples, audiologists and ENT doctors should optimize their phenotyping strategies for instance by using high frequency audiometry and multivariate questionnaire data (Muller et al., 2016; Schlee et al., 2017), initiate incentives to allocate a specific ICD-code for bilateral tinnitus, and start biobanking samples (Lopez-Escamez et al., 2016). Regarding the latter, since it is not custom for an ENT clinic to collect samples for DNA biobanking, guidelines should emerge to promote good practice (Fuller et al., 2017) and enable the creation of a large consortium to join efforts to decipher the genetic basis of tinnitus.

## Author Contributions

CC conceived the paper and prepared the table. CC co-wrote the paper with JL-E. AK and PS helped to develop the scientific arguments. All authors played a role in writing the manuscript and approved the final version.

## Conflict of Interest Statement

The authors declare that the research was conducted in the absence of any commercial or financial relationships that could be construed as a potential conflict of interest.

## Footnotes

1 http://tinnet.tinnitusresearch.net/
